# Impact of weekly case-based tele-education on quality of care in a limited resource medical intensive care unit

**DOI:** 10.1186/s13054-019-2494-6

**Published:** 2019-06-14

**Authors:** Pedja Kovacevic, Sasa Dragic, Tijana Kovacevic, Danica Momcicevic, Emir Festic, Rahul Kashyap, Alexander S. Niven, Yue Dong, Ognjen Gajic

**Affiliations:** 1 Medical Intensive Care Unit, University Clinical Centre of Republic of Srpska and Faculty of medicine, University of Banja Luka, Banja Luka, Bosnia and Herzegovina; 2 Clinical Pharmacy, University Clinical Centre of Republic of Srpska, Banja Luka, Bosnia and Herzegovina; 3 Department of Critical Care, Mayo Clinic, Jacksonville, FL USA; 4 Division of Pulmonary and Critical Care Medicine, Department of Medicine, Mayo Clinic, 200 First Street SW, Rochester, MN 55905 USA; 5 Department of Anesthesiology and Perioperative Medicine, Mayo Clinic, Rochester, MN USA

**Keywords:** Telemedicine, Case-based learning, Quality, Education, Checklist, Low resource, Intensive care

## Abstract

**Background:**

Limited critical care subspecialty training and experience is available in many low- and middle-income countries, creating barriers to the delivery of evidence-based critical care. We hypothesized that a structured tele-education critical care program using case-based learning and ICU management principles is an efficient method for knowledge translation and quality improvement in this setting.

**Methods and interventions:**

Weekly 45-min case-based tele-education rounds were conducted in the recently established medical intensive care unit (MICU) in Banja Luka, Bosnia and Herzegovina. The Checklist for Early Recognition and Treatment of Acute Illness (CERTAIN) was used as a platform for structured evaluation of critically ill cases. Two practicing US intensivists fluent in the local language served as preceptors using a secure two-way video communication platform. Intensive care unit structure, processes, and outcomes were evaluated before and after the introduction of the tele-education intervention.

**Results:**

Patient demographics and acuity were similar before (2015) and 2 years after (2016 and 2017) the intervention. Sixteen providers (10 physicians, 4 nurses, and 2 physical therapists) evaluated changes in the ICU structure and processes after the intervention. Structural changes prompted by the intervention included standardized admission and rounding practices, incorporation of a pharmacist and physical therapist into the interprofessional ICU team, development of ICU antibiogram and hand hygiene programs, and ready access to point of care ultrasound. Process changes included daily sedation interruption, protocolized mechanical ventilation management and liberation, documentation of daily fluid balance with restrictive fluid and transfusion strategies, daily device assessment, and increased family presence and participation in care decisions. Less effective (dopamine, thiopental, aminophylline) or expensive (low molecular weight heparin, proton pump inhibitor) medications were replaced with more effective (norepinephrine, propofol) or cheaper (unfractionated heparin, H2 blocker) alternatives. The intervention was associated with reduction in ICU (43% vs 27%) and hospital (51% vs 44%) mortality, length of stay (8.3 vs 3.6 days), cost savings ($400,000 over 2 years), and a high level of staff satisfaction and engagement with the tele-education program.

**Conclusions:**

Weekly, structured case-based tele-education offers an attractive option for knowledge translation and quality improvement in the emerging ICUs in low- and middle-income countries.

**Electronic supplementary material:**

The online version of this article (10.1186/s13054-019-2494-6) contains supplementary material, which is available to authorized users.

## Introduction

The World Health Organization defines telemedicine as “a health service in conditions where the distance is a critical factor, and it involves the use of information and communication technologies for the exchange of useful information about diagnosis, treatment and prevention of diseases and injuries, for research and continuous medical education of health workers, all in order to improve the health of individuals and their communities” [1]. Telemedicine in critical care has largely gone down the advanced E-ICU pathway to provide 24/7 intensivist support in high-income countries [2]. However, there are alternate paths within the scope of telemedicine suitable for wide array of health care settings, including “e-learning” or tele-education [3, 4].

Preliminary studies from Mayo Clinic investigators recently demonstrated that video-enabled remote simulation training based on a structured platform (CERTAIN: Checklist for Early Recognition and Treatment of Acute Illness) [5] can be a successful and efficient case-based learning method to disseminate clinical skills to critical care practitioners in diverse international settings [6, 7].

In low- and middle-income countries, tele-education may offer an attractive option to accelerate knowledge translation and address infrastructure barriers and limited opportunities for intensive care subspecialty training. Video-enabled, case-based discussions were chosen as a cost-efficient method to provide face-to-face education and reinforcement of the CERTAIN platform, which provides a standardized approach and checklist of common, evidence-based best critical care practices.

Our aim was to provide a longitudinal tele-education program and evaluate the impact of spaced, case-based learning and reinforcement on clinical practice and patient outcomes in a recently established intensive care unit in Bosnia and Herzegovina.

## Methods

### Context

Using a before-after cohort study design, the investigators evaluated the effect of a remote, video-enabled longitudinal case-based tele-education intervention on clinician satisfaction, ICU processes and procedures, clinical outcomes, and cost in the newly established medical intensive care unit (MICU) at the University Clinical Center of Republika Srpska. The MICU serves as a referral center for a region with 500,000 inhabitants and is currently the most advanced multidisciplinary MICU in Bosnia and Herzegovina. It should be noted that Bosnia and Herzegovina is in a post-war period and a country in transition. Currently, there are 8 ICU beds in the closed ICU with 8 specialists (internal medicine, pulmonary, neurology, anesthesiology), 3 physicians in training, 1 head nurse, and 19 bedside nurses. While not formally employed by the ICU, the MICU multidisciplinary team includes a pharmacist and 2 physical therapists available Monday through Friday.

### Tele-education intervention

**Fig. 1:**
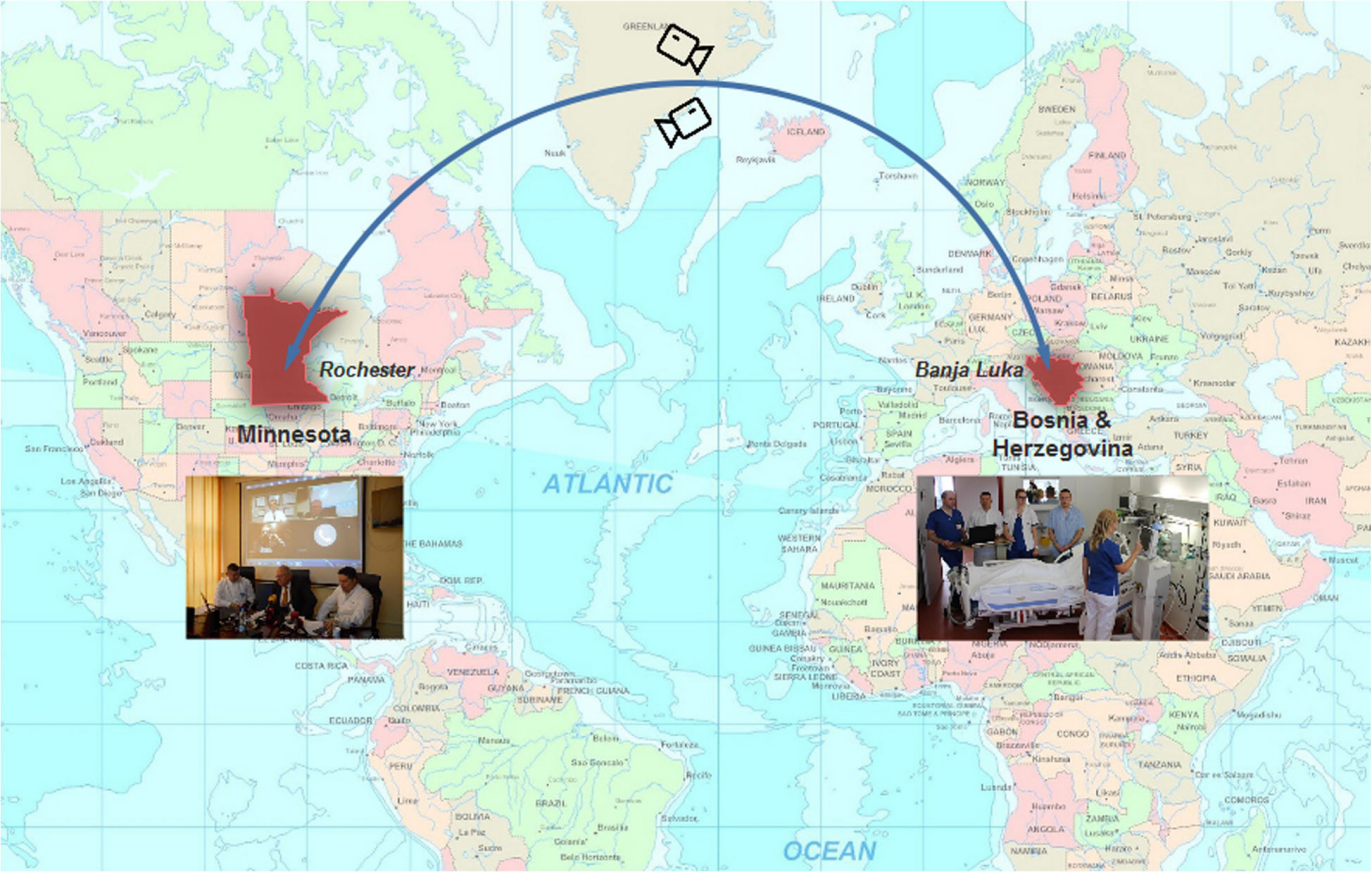
Weekly 45-min tele-education sessions occurred every Tuesday 2:30 pm, Europe, 8:30 am US central time using a secure two-way video connection

The tele-education intervention was conducted between January 2016 and December 2017 in cooperation with expert intensivists from Mayo Clinic in Rochester, Minnesota (OG), and Jacksonville, Florida (EF), who are both proficient in the local language and served as educators. The intervention involved a 45-min weekly engagement at a time that is convenient for both parties (Tuesday 2:30 pm, Europe, 8.30 am CST and 9.30 EST USA, Fig. 1). Learners included attending physicians, resident trainees, and nurses employed in the MICU. Discussion topics were selected to incorporate the breath of medical critical care, including specific organ failures and ICU syndromes, evidence-based interventions, and end of life care. During each tele-education session, a standardized patient presentation algorithm was used including the following outline.

#### Phase 1 (preparation)

Before each tele-education session, the host nation MICU physicians decided which active MICU patient would be presented. Selection was based on the severity and complexity of critical illness. Written patient summaries were emailed to the assigned educator prior to each session. The summary contained the reason for ICU admission, initial care, diagnostic and therapeutic procedures, and the patient’s condition in the previous 24 h.

#### Phase 2 (video session)

Learners presented the patient’s current clinical information followed by a diagnostic and treatment plan based on the CERTAIN Admission Checklist and the Rounding Checklist [5] to the assigned educator, followed by a group discussion of the clinical issues and diagnostic and management considerations. Learners included all MICU attending and resident physicians, as well the chief nurse and nurses involved in the care of the patient.

#### Phase 3 (evaluation)

All changes in the diagnostic and/or therapeutic plan were recorded. Electronic communication and expert evaluation continued beyond the tele-rounds ad hoc and as needed via e-mail.

Video sessions were performed via the ZOOM™ platform (www.zoom.us), which has been used as secure two-way video communication platform and is available as an application for PC Windows, Android, and iOS (Mac, Apple) operating systems. It allows for intuitive, easy to use real-time communication and secure sharing of multimedia content. The internet connection was supported by a wireless router in the MICU.

### Patient selection

The study included all patients admitted and treated in the MICU from January 1, 2015, to December 31, 2017. Patients admitted and treated in the MICU before the intervention (before 1/1/2016) served as a control group. Patient data were collected from the University Clinical Center information system and patients’ medical records, including baseline demographics, indication and duration of mechanical ventilation, use of vasopressors, and diagnosis-related groups (DRGs). DRGs were used as a measure of the baseline severity of illness. DRGs use a method of classifying acute hospital patients into groups with similar clinical features who require similar consumption of hospital resources.

### Study of intervention

Data required for DRG group determination include main diagnosis (ICD 10), additional diagnoses (complications and comorbidities ICD 10), main procedure, other procedures, age, gender, and admission source. Higher DRG values are associated with more severe clinical conditions. Main clinical outcomes included hospital and ICU length of stay, and unadjusted mortality. Data on MICU annual resource expenditures were obtained from the University Clinical Center Financial Office. Annual MICU spending is based on the consumption of pharmaceuticals, medical devices, and the cost of diagnostic and treatment services during a MICU stay. After each tele-education session, we also recorded changes in therapeutic and diagnostic algorithms, ad hoc correspondence, technical problems, and shortcomings. Changes in structure or processes were graded as “fully implemented,” “partially implemented,” or “not implemented.”

### Team

Staff satisfaction was measured using a survey instrument using questions based on the 5-point Likert scale that was distributed to all MICU employees at the of end of first year of the intervention. Sixteen providers (10 physicians, 4 charge nurses, and 2 physical therapists) who were employed in the hospital throughout the study period completed a semistructured evaluation of structure and processes in the ICU according to organ systems based on the CERTAIN platform [5].

### Statistical analysis

Baseline patient characteristics, treatment, and outcomes were compared among 3 years: (1) 2015, (2) 2016, and (3) 2017. Continuous variables were compared among the groups using the Mann–Whitney *U* test, Student *t*-test, and ANOVA, and categorical variables using the chi-squared test. Continuous data were summarized as medians with 25th to 75th percentiles (interquartile range, IQR). To evaluate the effect of the intervention on clinical outcomes, we performed a multivariate analyses taking into consideration severity of illness including age, DRG, vasopressor use, and invasive mechanical ventilation. The IBM SPSS 20 statistical program was used for all statistical analyses. The significance of the difference was tested (*p* <  0.01 and *p* <  0.05) for the monitored parameters.

### Ethical considerations

The study was approved by the local University Clinical Center Ethics Committee. All patient information was de-identified during tele-education discussions and e-mail correspondence to protect personal privacy during these interactions.

## Results

Forty-three tele-education sessions were conducted in 2016, and 39 in 2017 (82 total). Minimal technical issues (WiFi connection disruptions) were observed in 31 out of 82 sessions, and decreased over time.

Demographic and baseline characteristic of the patients included in this study and main outcomes are presented in Table 1. Age, gender distribution, and vasopressor support requirements were similar before and after intervention. DRG coefficients and the number of patients requiring invasive mechanical ventilation decreased in the period following the intervention (Table 1).

**Table 1:** Patients’ characteristics before and after implementation of tele-education program

Characteristics	Before	After		*P* value
Year	2015	2016	2017	
Number of patients	667	595	633	
Age	63.4 ± 16.2	62.2 ± 16.6	63.2 ± 0.37	0.238^a^
Male	365 (54.7%)	394 (66.2%)	387 (61%)	< 0.01^b^
Mechanically ventilated (invasive + noninvasive)	233 (34.9%)	162 (27.2%)	159 (25.1%)	< 0.01^b^
Vasopressor	246 (36.9%)	241 (40.5%)	244 (38.5%)	0.418^b^
Diagnosis-related group (DRG)	3.5 ± 0.25	3.2 ± 0.13	3.09 ± 0.24	< 0.01^a^

^a^ANOVA test

^b^Pearson *χ*^2^ test

Detailed changes in structure and processes according to each organ system (based on CERTAIN checklist) are presented in Table 2. Supporting quantitative data are provided in Additional file 1: Table S1, electronic data supplement. Structural changes among others included standardized admission and rounds, hand-washing dispensers and instructions, in-ICU physical therapy, assessment of antimicrobial sensitivity, point of care ultrasound, assessment and documentation of fluid balance, pharmacist review, closed ventilator suction, default lung-protective ventilator settings, and family presence. The process changes included daily sedation interruption, spontaneous breathing trials, restrictive fluid and transfusion, daily assessment of devices, and the use of prone position and neuromuscular blockade in severe ARDS. Less effective (dopamine, thiopental, aminophylline) or expensive (low molecular weight heparin, proton pump inhibitor) medications were replaced with more effective (norepinephrine, propofol) or cheaper (unfractionated heparin, H2 blocker) alternatives. The vast majority of changes were evaluated as “fully implemented.” Standardized nutrition, avoidance of polypharmacy, the use of beta blockers, and bone marrow biopsy were considered “partially implemented” by a minority of evaluators (25%).

**Table 2:** Care process changes in the University Clinical Center of Republika Srpska MICU after 2 years of weekly critical care tele-education

	Before	After
Central nervous system	Sedation interruption, neurologic assessment left to individual physician Thiopental primary choice for sedation Rare use of neuromuscular blockade, and only as (prolonged) infusion	Scheduled sedation interruption, neurologic assessment at least twice a day Propofol, midazolam primary sedative agents More frequent use of neuromuscular blockade (ARDS, intermittent or short-term use)
Cardiovascular system	Sporadic use of ultrasound to assess cardiac function Dopamine primary vasoactive medication Beta blocker use uncommon	Routine use of bedside ultrasound to assess cardiac function in all ICU patients Norepinephrine primary vasoactive medication Beta blockers frequently used for common indications
Respiratory system	No structured approach to mechanical ventilation, liberation Sporadic use of recruitment maneuvers, prone positioning No systematic approach to prevention, management of mechanical ventilation complications Frequent use of aminophylline Use of open aspiration systems Surgical tracheotomy No use of corticosteroids in pneumonia	Lung-protective mechanical ventilation in all ICU patients Regular use of restrictive fluid strategy, recruitment maneuvers, and prone positioning when indicated in ARDS Ventilator liberation protocol, with separation to noninvasive ventilation when indicated Routine use of ventilator bundle measures Use of aminophylline restricted to narrow indications Use of closed aspiration systems Percutaneous tracheotomy Use of corticosteroids in pneumonia with C-reactive protein > 150
Genitourinary system	No routine fluid balance calculations or volume assessment Liberal intravenous fluids, rare diuretic use Intermittent renal replacement only	Daily fluid balance calculation, documentation Dynamic assessment of volume status Restrictive intravenous fluid intake (enteral use preferred), regular diuretic use Establishing continuous renal replacement program
Gastrointestinal system	Nutrition administration left to individual physician Universal use of proton pump inhibitors (PPI) for stress ulcer prophylaxis	Standardized, early enteral nutrition with patient targeted needs. H2 antagonists for stress ulcer prophylaxis (PPIs reserved for upper GI bleeding from peptic ulcer disease)
Hematologic system	DVT prophylaxis with low molecular weight heparin (expensive) Liberal red cell transfusion (Hb < 8.5) Bone marrow biopsy not performed	DVT prophylaxis with unfractionated heparin (cost savings) Restrictive red cell transfusion (Hb < 7) Bone marrow biopsy performed when indicated
Infection prevention, management	Limited hand hygiene practices Frequent, long-term use of broad spectrum antibiotics Cultures and local antimicrobial sensitivity rarely used Regular tracking of multiple sepsis biomarkers (expensive)	Organized hand hygiene program Early empiric antibiotic treatment with rapid de-escalation Creation of local antibiogram to guide therapy selection
Skin and mucosa	No routine skin evaluation, with frequent complications	Routine skin, mucous membrane examination
Pharmacology	No input from hospital pharmacist	Regular pharmacist input, decreased medication administration and interactions using current guidelines, recommendations (UpToDate®) Routine antibiotic dosing adjustment based on renal, liver function
Devices	Device removal left to individual physician	Daily assessment for the need, removal of devices
Rehabilitation	Physical therapy consult left to individual physician	Physical therapist is an integrated member of ICU team on rounds, provides early mobilization
Treatment environment	Minimal, restricted family visitation	Continuous efforts to deliver patient-centered care Maximal family member engagement in patient treatment decisions, rehabilitation

Multiple changes in management were observed in 18 out of 82 sessions and included changes in medications, ventilator mode and settings, and/or diagnostic procedures. Management changes were more likely to occur in more complex patients and were seen in greater frequency early in the study period (total management changes were 118 in 2016 vs 89 in 2017). The same decreasing frequency of ad hoc e-mail correspondence (16 in 2016 vs 7 in 2017) was also seen over time.

ICU and hospital mortality, length of stay, and per-patient costs markedly decreased after the intervention (Additional file 1: Table S2, electronic data supplement). When adjusted for baseline DRG coefficients and the use of mechanical ventilation, the difference in mortality, length of stay, and cost remained significant. When adjusted for age, sex, mechanical ventilation, and vasopressors, tele-education remained associated with a significant decrease in hospital mortality (Additional file 1: Table S3) and shorter ICU and hospital length of stay (Additional file 1: Table S4, electronic data supplement).

A staff satisfaction survey was completed in 2016 and 2017 by all 10 participating physicians and nurses showing a universally high satisfaction with their interactions during the tele-education program.

## Discussion

The implementation of structured, weekly tele-education sessions in a MICU with limited resources was feasible, well received, and associated with a significant improvement in clinical outcomes and associated costs of care. The results demonstrate that the essential elements of high-quality critical care are not expensive and can be implemented outside high-resource settings.

Although methodologically limited and inconsistent, most telemedicine studies in the ICU have reported improved clinical outcomes including mortality, reduction of the average length of hospitalization, and overall treatment costs [8–12]. These observed benefits are likely at least in part explained by education of bedside providers and reinforcement of best evidence-based practices that was the key focus of our intervention (i.e., tele-education). While our intervention is clearly not comparable to a comprehensive telemedicine program, we feel that the results of our study suggest tele-education may offer a favorable alternative in resource-limited settings. The cost of advanced tele-ICU systems is substantial, due to the installation, staffing, and maintenance of the command center and complicated software platforms. Yet, the evidence for benefit is limited. In contrast, our intervention used a secure but affordable video-conference platform and focused on an efficient expert knowledge transfer in the form of a case-based, concise tele-education session. We have shown that it is possible to make significant improvements with modest investments in time and technology. These results are encouraging and provide proof of concept to expand this program to other emerging ICUs in our region and in other low- and middle-income countries using a train-the-trainer model.

Our results are clearly influenced by the consistent use of a standardized approach to the evaluation and treatment of critically ill patients using the CERTAIN Admission Checklist and the CERTAIN Rounding Checklist, which both reduced practice variation and the risk of medical error. This standardized approach has become part of the practice in our ICU, leading to the reduction of unnecessary diagnostic, therapeutic procedures, and treatments in accordance with evidence-based recommendations for critical care practice. Physician and nursing staff in our MICU were very accepting and satisfied with this tele-education program, due in no small part to the interprofessional engagement and leadership support in our MICU that are commonly recognized to be essential components of a successful change management. The hospital leadership support was essential and was reinforced by the CERTAIN program influence in obtaining certification (ISO9001:2015) with our ICU becoming a referral medical ICU for an area covering 500,000 people.

To analyze the actual effect of this tele-education program on mortality and average hospitalization, we compared processes, treatments, and patient outcomes in 2015, 2016, and 2017. Although the general demographics of our population remained unchanged, we found the number of mechanically ventilated patients decreased sequentially. This could be explained by increased use of noninvasive ventilation in our setting, including its application in the step down unit of our hospital. We also recorded a decrease in the DRG coefficient during all 3 years, which may also be a reflection of decreased mechanical ventilated days or a reduction in complications due to adoption of evidence-based critical care practices (i.e., restrictive fluid resuscitation strategies).

Our study results do include several limitations that potentially impact the generalizability of these findings. We used a DRG coefficient and the use of vasopressors and invasive mechanical ventilation as markers of severity of illness. While more complex severity of illness scores such as Acute Physiology and Chronic Health Evaluation (APACHE) have not been validated in low resource settings [13], the absence of a standardized case-mixed adjustment is a principal methodologic limitation. In the before-after design, it is impossible to attribute the observed changes in practice and outcomes to the tele-education as opposed to natural trends. While no other quality improvement, educational interventions, or staffing changes occurred during the study period, multiple additional unmeasured factors could have influenced the observed results. The decrease in the use and duration of invasive mechanical ventilation can in part be explained by the emphasis on weaning and increased use of noninvasive ventilation after the intervention.

Although the evidence for the efficacy of the CERTAIN checklist per se is limited at this time, CERTAIN content of the CERTAIN content is non-controversial and is based on current recommendation by practice guidelines from critical care societies [14].

Unfortunately, formal educational outcomes were not collected. However, indirect proof of knowledge gained is the fact that five physicians subsequently passed independent critical care subspecialty exam. With regard to cost analysis, our hospital database does not keep an accurate account of per-patient spending. Therefore, we calculated the total spending based on the consumption of pharmaceuticals and medical devices and the cost of diagnostic and other services for the entire ICU before and after the intervention. Finally, the study was conducted in a single center with motivated staff and leadership support, which may not be present in all limited resource settings.

## Conclusions

In conclusion, weekly 45-min, tele-education case-based learning sessions utilizing the CERTAIN platform were effectively adopted into the medical ICU practice in a limited resource setting and were associated with the improvement of clinical outcomes and reduced costs. Low-cost tele-education interventions can serve as a good model for educating health workers and improving care and outcomes of critically ill patients in low- and middle-income countries.

## Additional file

Additional file 1: **Table S1.** Granular data pertinent to changes in practice before and after tele-education intervention. **Table S2**. Multivariate linear regression for length of stay in the ICU. **Table S3**. Multivariate linear regression for length of stay in the hospital. **Table S4**. Multivariate linear regression for length of stay in the hospital. (DOCX 18 kb)

## Data Availability

The data that support the findings of this study are available from the corresponding author upon reasonable request.

## Abbreviations

CERTAIN: Checklist for Early Recognition and Treatment of Acute Illness
DRG: Diagnosis-related groups
ICU: Intensive care unit
MICU: Medical intensive care unit
