# Supplementary material for: Impact of weekly case-based tele-education on quality of care in a limited resource medical intensive care unit
Source: Crit Care. 2019 Jun 14;23:220. doi: 10.1186/s13054-019-2494-6 (PMC6567671; doi:10.1186/s13054-019-2494-6)
Supplement: Supplementary file 1 — Table S1. Granular data pertinent to changes in practice before and after tele-education intervention. Table S2. Multivariate linear regression for length of stay in the ICU. Table S3. Multivariate linear regression for length of stay in the hospital. Table S4. Multivariate linear regression for length of stay in the hospital. (DOCX 18 kb) [file 13054_2019_2494_MOESM1_ESM.docx]

**Additional file 1: Table S1** Granular data pertinent to changes in practice before and after tele-education intervention

|  | Before | After | | Data Source |
| --- | --- | --- | --- | --- |
| Year | 2015 | 2016 | 2017 |  |
| Propofol, orders | 2700 | 2090 | 4100 | Pharmacy |
| Neuromuscular blocking agents, orders | 60 | 530 | 410 | Hospital |
| Dopamine, orders | 1166 | 1130 | 300 | Pharmacy |
| Norepinephrine, orders | 472 | 980 | 1250 | Pharmacy |
| Amynophylline, orders | 180 | 250 | 130 | Pharmacy |
| H2 blockers, orders | 2330 | 3502 | 2740 | Pharmacy |
| Low molecular weight heparin, orders | 684 | 630 | 340 | Pharmacy |
| Antibiotic, orders | 983 | 901 | 729 | Pharmacy |
| Meropenem, orders | 831 | 621 | 575 | Pharmacy |
| All medication orders | 4362 | 3987 | 3417 | Pharmacy |
| Chest radiographs, referrals | 683 | 615 | 582 | Administration |
| Laboratory tests | 4362 | 3987 | 3417 | Administration |
| Average duration of central venous catheter use in patients with sepsis, days (n=150) | 6.2 | 5.7 | 4.2 | Chart review |
| ICU acquired infection | 33% | 30% | 12.5% | Administration |

**Table S2** Outcomes before and after tele-education intervention

|  | Before | After | | P value |
| --- | --- | --- | --- | --- |
| Year | 2015 | 2016 | 2017 |  |
| ICU mortality | 237 (42.6%) | 171 (30.8%) | 148 (26.6%) | < 0.01^b^ |
| Hospital mortality | 341 (51.1%) | 300 (50.4%) | 276 (43.5%) | p= 0.01^b^ |
| Length of stay (days) | 8.3 ±0.72 | 7.1±0.54 | 3.6±0.62 | < 0.01^a^ |
| Annual consumption (medications, equipment, services) ($) | 497,417 | 278,057 | 293,176 |  |
| Annual per patient consumption (medications, equipment, services) ($) | 746 | 467 | 463 |  |

^a^ANOVA test

^b^Pearson χ^2^ test

**Table S3** Multivariate linear regression for length of stay in the ICU

| Characteristics | Estimate | Lower 95% | Upper 95% | P value |
| --- | --- | --- | --- | --- |
| Tele-education intervention | -2.97 | 0.07 | -39.91 | <0.01 |
| Age | -0.01 | 0 | -2.74 | <0.01 |
| Female | 0.51 | 0.09 | 5.88 | <0.01 |
| Invasive mechanical ventilation | -0.04 | 0.1 | -0.41 | 0.68 |
| Vasopressors | 0.05 | 0.07 | 0.69 | 0.49 |
| Diagnosis related group (DRG) | 1.2 | 0.90 | 1.51 | <0.01 |

**Table S4** Multivariate linear regression for length of stay in the hospital

| Characteristics | Estimate | Lower 95% | Upper 95% | P value |
| --- | --- | --- | --- | --- |
| Tele-education | -1.37 | -3.09 | 0.33 | 0.12 |
| Age | -0.029 | -0.08 | 0.03 | 0.28 |
| Female | -1.34 | -2.93 | 0.26 | 0.10 |
| Mechanical ventilation | 1.47 | -0.35 | 3.28 | 0.11 |
| Vasopressors | 2.73 | 1.40 | 4.05 | <0.01 |
| Diagnosis related group (DRG) | -0.51 | -3.43 | 2.44 | 0.73 |
